# Calcium Phosphates as Delivery Systems for Bisphosphonates

**DOI:** 10.3390/jfb9010006

**Published:** 2018-01-13

**Authors:** Adriana Bigi, Elisa Boanini

**Affiliations:** Department of Chemistry “G. Ciamician”, University of Bologna, 40126 Bologna, Italy

**Keywords:** hydroxyapatite, bisphosphonates, octacalcium phosphate, β-tricalcium phosphate, functionalization, alendronate, zoledronate

## Abstract

Bisphosphonates (BPs) are the most utilized drugs for the treatment of osteoporosis, and are usefully employed also for other pathologies characterized by abnormally high bone resorption, including bone metastases. Due to the great affinity of these drugs for calcium ions, calcium phosphates are ideal delivery systems for local administration of BPs to bone, which is aimed to avoid/limit the undesirable side effects of their prolonged systemic use. Direct synthesis in aqueous medium and chemisorptions from solution are the two main routes proposed to synthesize BP functionalized calcium phosphates. The present review overviews the information acquired through the studies on the interaction between bisphosphonate molecules and calcium phosphates. Moreover, particular attention is addressed to some important recent achievements on the applications of BP functionalized calcium phosphates as biomaterials for bone substitution/repair.

## 1. Introduction

The main inorganic components of the hard tissues of vertebrates are calcium orthophosphates (CaPs). In particular, the mineral phase of bone is described as a calcium phosphate similar to synthetic hydroxyapatite (Ca_10_(PO_4_)_6_(OH)_2_, HA), although it differs from HA in several aspects, including poor crystallinity, small crystal dimensions and non-stoichiometry due to the presence of a number of associated foreign ions [1]. Overall, the average age of the population in developed countries is continuously increasing with consequent increase of age-related problems, including musculoskeletal disorders. The problem requires the development of suitable materials for the substitution and repair of damaged tissues. A successful biomaterial should be able to interact with and bond to the surrounding biological tissue, a requirement which can be best achieved the more the synthetic material resembles the biological one in terms of structure, composition, morphology and functionality [2]. On this basis, CaPs are widely employed in the preparation of biomaterials for bone tissue substitution and repair. Although HA is the calcium orthophosphate more similar to the mineral phase of bone, the number of biocompatible and bioactive CaPs includes several members, such as octacalcium phosphate (Ca_8_(HPO_4_)_2_(PO_4_)_4_·5H_2_O, OCP), tricalcium phosphate, both as α-TCP and β-TCP, dicalcium phosphate in the dihydrate (CaHPO_4_·2H_2_O, DCPD) and anhydrous (CaHPO_4_, DCPA) forms, and tetracalcium phosphate (Ca_4_(PO_4_)_2_, TTCP). OCP is considered the precursor phase of biological apatite and has been detected as an intermediate phase in the precipitation of synthetic HA; α-TCP and TTCP are widely employed for the preparation of calcium phosphate bone cements; β-TCP is of great interest for its excellent biodegradability [1,3].

The biological performance of CaPs can be significantly improved and tailored through functionalization with ions, molecules, macromolecules, drugs and growth factors, in order to get suitable materials for the local administration of therapeutic agents [4]. In particular, functionalization of calcium phosphates with bisphosphonates is attracting increasing interest. Bisphosphonates (BPs) are a class of molecules widely and successfully utilized for the treatment of pathologies associated to disorders of bone metabolism, including osteoporosis, Paget’s disease, fibrous dysplasia and multiple myeloma, as well as bone metastases from solid tumors [5,6,7].

However, long-term use of these drugs, especially in high doses, has been reported to cause a number of undesirable side effects due to over-suppression of bone resorption [8,9]. Alternative modes of administration, such as the use of systems for BP local release at specific bone sites, have been suggested with the aim to reduce the doses of systemic administration and related adverse side effects. The proposed systems include nanoparticles, liposomes, implants, transdermal systems, coatings, cements and scaffolds, with particular attention to calcium phosphate-based biomaterials [9,10]. This paper reviews the most recent and interesting studies on the interaction between bisphosphonates and calcium phosphates, as well as on the potential applications of CaPs as delivery systems for the local administration of BPs.

## 2. Bisphosphonates

### 2.1. Classification and Structure

**Figure 1:**
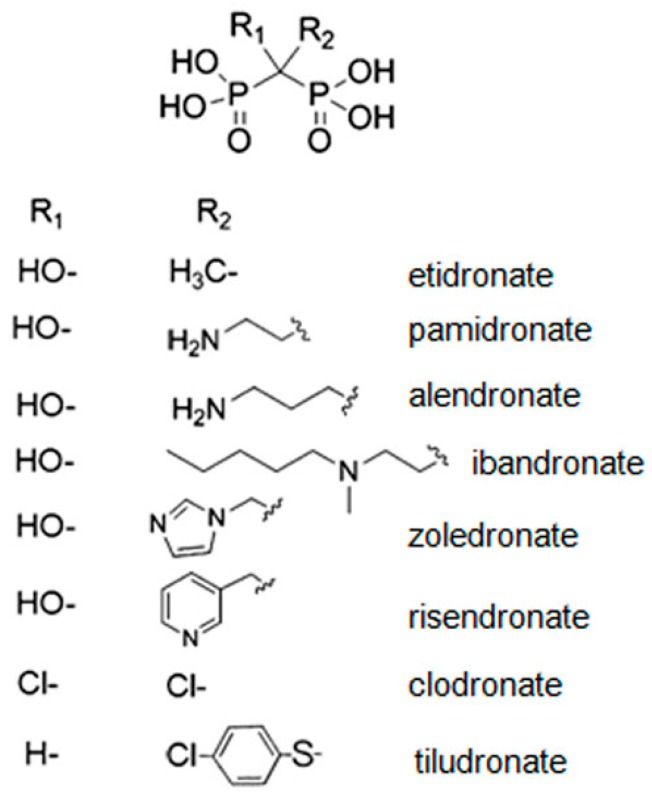
Molecular structure of Bisphosphonates. Modified with permission from reference [13] Copyright (2012) American Chemical Society.

Bisphosphonate molecules are characterized by a common backbone structure of P-C-P, where each P bound to the central carbon atom is a phosphonate group. The P-C-P bridge is resistant both to chemical and enzymatic hydrolysis [11]. The two phosphonate groups are essential for the binding to the mineral phase of bone, as well as for cell-mediated antiresorptive activity of these drugs. The mechanism of inhibition of hydroxyapatite crystal growth can be ascribed to calcium complexation of bisphosphonates. In fact, the two phosphonate groups can interact with calcium atoms on the HA surface through a bidentate chelation of deprotonated oxygen atoms thanks to the O-O distance of these anions, which is in the same range as the Ca-O mean distances in HA [12]. Two covalent side chains, R1 and R2, complete the tetravalence of the carbon atom (Figure 1).

The presence of OH as an R1 substituent provides a tridentate binding to calcium [11]. Bisphosphonates are classified as non-nitrogen and nitrogen BPs (N-BPs) on the basis of the absence and presence, respectively, of a nitrogen atom in their R2 side chain. Nancollas et al. (2006) used a constant composition potentiostatic method to demonstrate the influence of zeta potential on bisphosphonate binding activity to synthetic HA. The results showed that BP binding activity increases in the following order: Clodronate < etidronate < risendronate < ibadronate < alendronate < zoledronate. This rank was confirmed by the results of a chromatographic study [14]. Slightly different rank orders were found using different methods [15]. However, there is general agreement on the greater binding affinity of BPs with a nitrogen atom in their R2 side chain than non-nitrogen containing BPs. Potential interaction of N-BPs with the HA surface was simulated using 3D computational modeling [11,16]. The results of these studies indicate that nitrogen can bind to the hydroxyl group on the HA surface forming N-H-O hydrogen bonds. Optimal binding has been calculated to occur at a bond angle ≥125° and a bond distance of about 3 Å. The molecular structure of alendronate completely fulfills these requirements, which are satisfied also by zoledronate, whereas the orientation of nitrogen in risedronate is not suitable for hydrogen bonding [11].

### 2.2. Mechanism of Action

**Figure 2:**
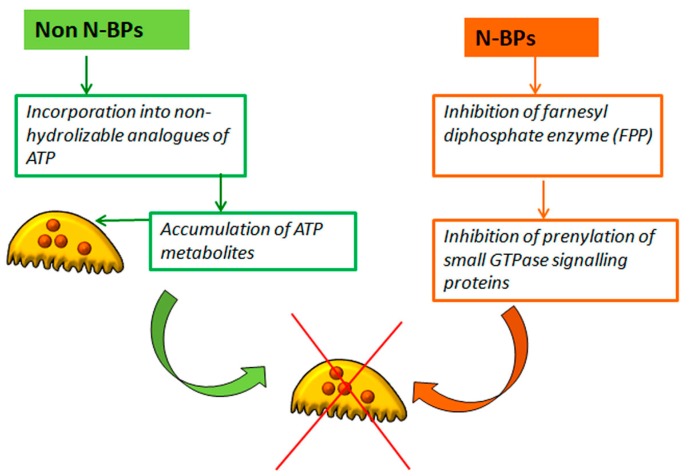
Bisphosphonates inhibit osteoclast activity via different mechanisms. Non N-BPs: Non-nitrogen bisphosphonates; N-BPs: Nitrogen-bisphosphonates. See text for details.

Non-nitrogen and nitrogen BPs (N-BPs) display different mechanisms of action (Figure 2). Non-nitrogen bisphosphonates are metabolically incorporated into non-hydrolysable analogs of adenosine triphosphate. Accumulation of resulting metabolites within osteoclast inhibits their function and may lead to osteoclasts apoptosis [5]. On the other hand, N-BPs interfere with specific metabolic reactions, in particular in the mevalonate pathway, which leads to the synthesis of cholesterol and other sterols. Among the several enzymes, which are inhibited by N-BPs in the mevalonate pathway, farnesyldiphosphate synthase (FPPS) plays a key role. The inhibition of this enzyme hinders the prenylation of small GTPase signaling proteins, thus preventing the correct function of these proteins which regulate a number of cell processes important for osteoclast function, such as morphology, cytoskeletal arrangement, membrane ruffling and formation of actin rings. At high concentrations, N-BPs can induce osteoclast apoptosis, blocking bone resorption [5,10,17].

The same molecular mechanism has been indicated to contribute to the direct antitumor effect of N-BPs [18,19]. Moreover, these drugs display also indirect anticancer activities preventing angiogenesis and disturbing vascularization of primary tumors and premetastatic niches. In fact, N-BPs regulate proliferation and activity of macrophages and endothelial cells and modulate the immune system via activation of γδ T cells [9,20]. It follows that BPs are not only the most applied therapy for preventing and treating osteoporosis, but are also useful to treat multiple primary bone tumors and bone metastases associated to solid tumors and osteolytic lesions from multiple myeloma [21,22,23].

Generally speaking, BPs’ influence on osteoclasts has been reported to include: inhibition of the recruitment and differentiation of osteoclast precursors, inhibition of resorption by mature osteoclasts, promotion of apoptosis of mature osteoclasts [24,25,26]. Suppression of osteoclast activity, either directly or mediated via osteoblasts [27], implies reduction of the number of active sites of bone remodeling with consequent reduction of bone loss. It is worthwhile to mention that BPs’ influence on bone turnover varies significantly on different bone sites; moreover turnover suppression depends on treatment time and on the type of bisphosphonate molecule [28].

In vitro studies on the effects of BPs on osteoblasts report an increase of proliferation and promotion of differentiation, as well as inhibition of proliferation and activity. The presence of apparently contradictory data can be explained by the dose dependent influence of BPs on osteoblasts: it has been found that at low concentrations (10^−9^–10^−6^ M) they promote proliferation and activity, whereas at relatively high concentrations (>10^−5^ M) they exert an inhibitory role [29]. Moreover, at low concentrations BPs have been reported to prevent glucocorticoid-induced apoptosis of osteoblasts and osteocytes [30].

### 2.3. Side Effects of BPs Therapy

The clinical efficacy of BPs is well demonstrated: they significantly inhibit bone loss and their long term use leads to excellent antifracture action, which persists after treatment suspension [31,32,33,34]. However, BPs have unfavorable pharmacokinetics, since most of systemic administered BPs binds to the skeleton or is cleared via renal filtration [35]. BPs exhibit a poor oral bioavailability: in the gastrointestinal tract they chelate calcium and form complexes with consequent reduction of adsorbance. Bioavailability increases on increasing dosage, while it is reduced by food and divalent cations [6]. Important side effects of these drugs include osteonecrosis of the jaw and atypical subtrochanteric femoral fractures [36,37,38]. The risk of infections and osteonecrosis of the jaw seems to be higher after long term use, especially in intravenous formulations, and is more frequently associated to treatments with high doses of the drugs, as in the case of oncologic patients [36,39,40]. Dental extraction in elderly multiple myeloma patients has been listed among possible risk factors [41]. The occurrence of non-traumatic fractures at atypical sites, as the subtrochanteric diaphysis of the femur reported by some studies, has been related to severe suppression of bone turnover due to long term treatment with BPs [9,37,42]. Moreover, a recent study of postapoptotic mineralization of osteocyte lacunae in bisphosphonate exposed human bone revealed the presence of magnesium whitlockite, Ca_18_Mg_2_(HPO_4_)_2_(PO_4_)_12_, crystals. The consequence of this deposition is an alteration of the nanomechanical properties due to the presence of localized areas of increased stiffness [43].

## 3. Calcium Phosphates as Delivery Systems for Bisphosphonates

Most of the studies on the functionalization of CaPs with BPs have been performed using hydroxyapatite as the inorganic phase. A significantly smaller number of studies have investigated the interaction of BPs with other CaPs, namely β-TCP and OCP [4]. Two main methods have been applied to synthesize BPs-CaPs: Chemisorptions from solution and co-precipitation in aqueous medium [4,9]. Co-precipitation generally requires modification of the usual strategies of synthesis because of the great affinity of BPs for calcium ions, which leads to the precipitation of calcium bisphosphonate salts [44]. On the other hand, BP loading onto CaPs through chemisorptions is an easier route, but usually yields less amount of adsorbed drug than co-precipitation [45]. Moreover, chemisorptions should provide a weaker interaction between BPs and CaPs than co-precipitation and, as a consequence, a faster drug release in solution.

### 3.1. Functionalization through Co-Precipitation

In the literature it is possible to find a few papers about co-precipitation methods for the preparation of CaPs functionalized with BPs. A reason for this is the experimental trouble due to the high affinity of BPs for calcium ions in solution: when co-present in the reaction solution BPs and phosphate ions will compete for calcium and, eventually, phosphonate groups will show a higher affinity and bind to calcium. This event will lead to the sequestration of calcium from solution and the precipitation of a calcium-BP compound, which will hinder precipitation of the CaP phase. This is the case of the direct preparation of HA-alendronate composite crystals. It has been shown that the affinity of alendronate for calcium is so high that when a classical method of synthesis of HA in aqueous medium is carried out in the presence of alendronate by adding alendronate and phosphate ions at the same time to calcium-containing solution, part of the product is constituted of amorphous material [44]. The presence of amorphous aggregates is clearly detectable also from the morphological evaluation of the obtained mixture by means of TEM and ED, where both apatitic crystalline particles and spherical-shaped amorphous particles can be recognized. Further chemical analysis revealed that the amorphous phase had the chemical stoichiometry of calcium alendronate monohydrate.

The precipitation of HA as a unique crystalline phase where alendronate is incorporated into apatitic crystals up to 7 wt % could be obtained after a slight modification of the synthesis procedure, that is by drop-wise addition of the alendronate solution to the reaction medium immediately after finishing the addition of ammonium phosphate [44]. In this way alendronate can bind to calcium, when most of calcium has already interacted with phosphate and HA crystal nucleation and growth has already started. As a result, the composite crystals, according to the powder structural refinements, display just a slight structural disorder with no significant variation of the atomic positions, occupancy factors, and thermal parameters with respect to pure HA. Furthermore, the samples do not contain an appreciable amorphous fraction, also according to the morphological analysis. When in vitro cultured on crystalline alendronate functionalized HA, osteoblasts display increased values of alkaline phosphatase activity, collagen type I, and osteocalcin production, as well as a significant decrease of matrix metalloproteinases (MMP-1 and MMP-13) production, with respect to pure hydroxyapatite nanocrystals. Moreover, the inhibitory effect of alendronate on osteoclast proliferation showed that alendronate is able to influence bone cells even in composite nanocrystals [13]. Release tests showed a very good correlation between the cumulative amount of released alendronate and the square root of time, suggesting that alendronate release kinetics follows a Higuchi model [46]. According to the Ritger–Peppas model, the kinetics of the release involves anomalous (non-Fickian) transport which means that the drug release involves several processes: diffusion, desorption or surface erosion. The observed sustained release up to 30 days could be relevant for applications as drug-delivery system [46]. In vivo studies showed that alendronate functionalized HA improves the biological performance with a dose response effect in comparison with HA, representing a promising strategy especially in osteoporotic patients with high risks of spinal fusion failure [47].

**Figure 3:**
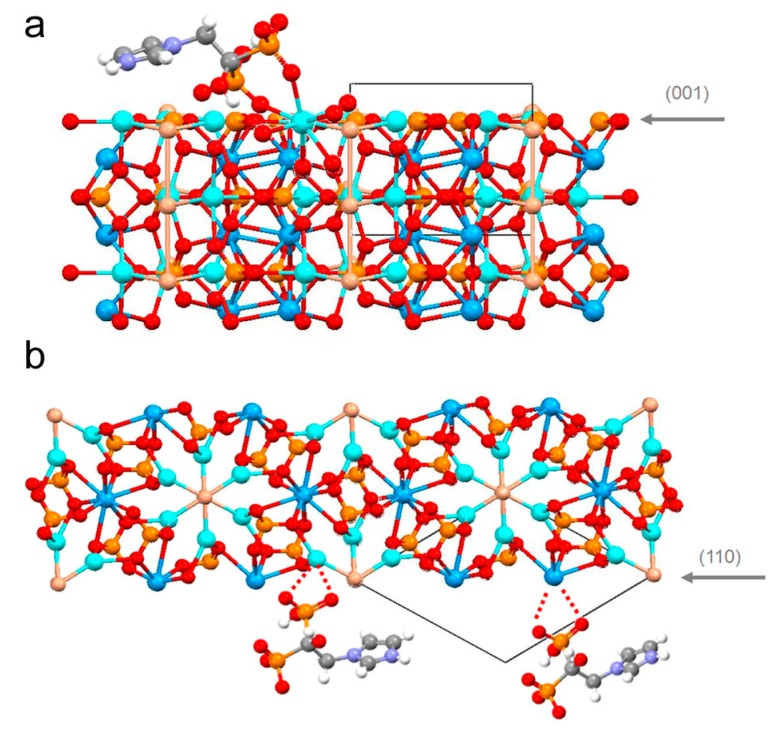
Sketch of the possible interaction of zoledronate with hydroxyapatite structure: (**a**) Zoledronate coordination of a Ca(2) atom at the (001) surface; (b) coordination at the (110) plane: the formation of possible N-H-O bonds is indicated. The coordination of Ca atoms in plot (**b**) is incomplete because the layer was reduced for reason of clarity. Reprinted from reference [48] Copyright (2012), with permission from Elsevier.

Application of a co-precipitation method to zoledronate functionalized HA did not show any particular synthetic problems and zoledronate could be incorporated up to about 7 wt % into HA crystals following a classical method of synthesis of HA in aqueous medium by simultaneous addition of zoledronate and phosphate ions to calcium-containing solution [45,48]. Zoledronate incorporation causes a decrease of the HA crystalline domains size and crystal dimensions, whereas the specific surface area increases. The structural refinement indicates no significant variation with respect to pure HA, and a good structural fit, which might contribute to justify why the synthesis of HA-zoledronate composite materials is relatively easy with respect to that of HA-alendronate. In fact, zoledronate and hydroxyapatite show similar calcium ion coordination geometry and possibility of hydrogen bonds, which results in a preferential interaction of zoledronate molecules with the hydroxyapatite faces parallel to the c-axis direction (Figure 3).

In vitro co-culture results show positive influence of zoledronate on osteoblast proliferation and differentiation even when the drug was incorporated into the hydroxyapatite crystals. Furthermore, zoledronate not only displays a greater affinity than alendronate for HA structure, but exerts an even greater influence on osteoclast apoptosis [48]. The high stability of HA-zoledronate interaction allows also the direct synthesis of strontium-hydroxyapatite-zoledronate crystals, where strontium is partially substituted to calcium in the structure of HA [49]. Strontium administration has been suggested as a therapeutic option to BPs, since the strontium ion is involved in bone metabolism, and strontium ranelate administration has been shown to reduce fracture risk in osteoporotic patients [50]. Different combinations of strontium and zoledronate amounts provoke a decrease of the length of the size of the perfect crystalline domains and an expansion of the crystal lattice of HA, mainly due to the substitution of calcium with the bigger strontium ion. The simultaneous presence of strontium and zoledronate shows their additive effect on the osteoblast response enhancing osteoblast viability and activity, whereas it reduces osteoclast proliferation and differentiation [49].

Multifunctionalization of HA has also been obtained by coupling alendronate and quercetin, a flavonoid with anti-oxidant properties. Loading of quercetin up to about 5 wt % on HA-alendronate composite crystals does not provoke any significant structural and morphological modifications, but the new materials exhibit relevant anti-oxidant properties, in agreement with their high radical scavenging activity [51]. In vitro tests were performed using an oxidative stress model induced by H_2_O_2_ in the culture, which greatly affects osteoblast viability: in the presence of quercetin and alendronate multifunctionalized HA osteoblast grew regularly, whereas the materials displayed a downregulatory effect towards co-cultured osteoclasts.

Also octacalcium phosphate-BPs composite materials can be obtained by direct synthesis. In the presence of zoledronate, as well as of alendronate, it is possible to obtain a single crystalline phase up to a content of 3.5 wt % and 5.2 wt %, respectively [52]. Both BPs provoke a reduction of the crystal dimensions of OCP and are able to stabilize OCP, preventing its hydrolysis into HA. Release tests showed that alendronate containing samples display an initial burst release of the BP in solution, whereas zoledronate is not appreciably released. In vitro tests in a triculture model showed that zoledronate and alendronate maintain their antiresorptive and anti-tumor properties even when incorporated into OCP [52].

### 3.2. Functionalization through Chemisorptions

Incubation of CaPs in BP solutions is an easy and common way to obtain functionalization, due to the high affinity of BP molecules for calcium ions exposed on the surface of the inorganic crystals. Many works have been devoted to clarifying the interactions that occur, also comparing different BPs. The adsorption can be followed by zeta potential measurements thanks to different behavior in protonation of BPs side chain moieties. At pH 5.0 and in the absence of any bisphosphonate, the zeta potential value for HA is 9.6 mV, whereas it becomes more positive as a function of concentration for all the examined BPs (with the exception of clodronate) [53]. Loading of zoledronate on β-TCP or calcium deficient apatites can also be monitored through ^31^P NMR spectra of the solutions that clearly show the signals due to the presence of zoledronate together with an additional peak at high field corresponding to phosphate species released from the solid substrate during the reaction [54]. Interestingly, this analysis revealed that most of the bisphosphonate was incorporated onto the CaP, with a value twice as high as the released phosphate concentration. ^31^P CP-MAS solid state NMR spectra and SEM morphological analysis of the modified compounds indicated two different association modes as a function of the CaP support and/or the initial concentration of the zoledronate solution. β-TCP is able to promote the formation of zoledronate-containing crystals on the surface of the CaP, which later were found to be metastable, leading to a biologically active calcium zoledronate compound [55]. On the other hand, calcium deficient apatite behavior seems to depend on zoledronate concentrations and could lead to chemisorption of the drug thanks to displacement of inorganic phosphate due to PO_3_ for PO_4_ exchange. A quantitative plateau in zoledronate uptake is reached at about 10 wt %, which corresponds to the saturation of the support surface [56]. The idea that chemisorption process occurs through a phosphate-phosphonate exchange on the surface of inorganic crystals has been supported through solid state NMR spectroscopy by other studies, which also extended this hypothesis of mechanism to other BPs such as alendronate, pamidronate and risedronate [57] and led to the formulation of a simple mathematical model of zoledronate interaction with HA surface [58]. The local release of zoledronate adsorbed onto HA materials has been shown to be able to prevent in vitro osteoclastic resorption without any adverse effects on osteoblastic cell [59].

Besides the important role of the two phosphonate groups, also the contribution of the substituent R1 and R2 groups to the binding affinity has been explored using NMR. The binding affinity to HA of etidronate, medronate, clodronate, alendronate and 14 their phosphoesters was determined after 1 h of reaction. Clodronate had poor affinity for HA, when compared to other BPs, because it lacks an hydroxyl group which takes part in the binding process. Furthermore, it was found that the ability of BPs to bind decreases when the number of ester groups increases and depends also on the location of the ester groups [60].

The results of a series of studies carried out on the interaction of BPs with hydroxyapatite using FTIR, Raman and solid NMR techniques, allowed them to propose a binding mechanism involving both phosphate substitution and interaction with calcium groups on the HA surface. For tiludronate, the mechanism was suggested to occur in two steps: (1) interaction with Ca^2+^ ions on the apatitic surface involving partial or total deprotonation of phosphonate groups; (2) interaction of the released protons with surface hydrogen phosphate groups, yielding release of H_2_PO_4_^−^ ions [61]. A third step, involving nitrogen bonding of a bisphosphonate molecule with an OH group on the apatite surface and the formation of an N-H-O bond, was hypothesized in the case of the N-BP risedronate [62]. Moreover, the adsorption parameters were found to be related to the experimental conditions, such as pH and temperature, and to the surface properties of the apatitic supports, in particular to the presence of a surface hydrated layer [61,62,63].

BPs adsorption and release can be modulated by using functionalized CaPs crystals as supporting materials. The profile of risedronate interaction with pure HA, zinc-substituted HA (ZnHA, Zn content about 8 atom %) and poly-ethylenimine-functionalized hydroxyapatite (HAPEI, PEI content about 2.5 wt %) displays Langmuir isotherms of adsorption for all the supports. On the contrary, risedronate release was directly related to the physicochemical characteristics of the support, revealing that risedronate adsorption on HA and ZnHA crystals occurs through chemisorption on apatitic phase, whereas also physisorption occurs on HAPEI inorganic-organic support, due to the presence of PEI on the surface of the crystals. As a result, the total amount of risedronate released from HA and ZnHA was smaller (about 5 and 9% respectively of the initial content) than from HAPEI (about 14%), where both mechanisms of adsorption contribute to modulate BP release [64]. The sinergy of different functionalizations may be useful in materials applications. Strontium-substituted HA (strontium content of about 10 atom %) was loaded with zoledronate through soaking in solution and the resulting composite nanoparticles used to treat ovariectomized rats with a single intravenous dose. The treatment provoked significant improvements in trabecular bone microarchitecture and mechanical strength as compared to the separate use of zoledronate or strontium-substituted HA [65].

## 4. Applications

### 4.1. CaPs Bone Cements

Calcium phosphate bone cements (CPCs) represent the biomimetic alternative to the most widely employed material for implant fixation that is poly-methylmetacrylate (PMMA). CPCs are biocompatible, bioactive and osteogenetic systems [66,67]. Their composition includes a powder, constituted of one or several calcium phosphates, and a liquid phase: mixing the two components yields a workable paste that stiffens during setting and hardening into a solid phase. Differently from the polymeric reaction of methylmetacrylate, the hardening reaction is not exothermic. The vast variety of compositions proposed for CPCs leads to just two possible final products: hydroxyapatite and brushite (DCPD), which is a metastable phase and eventually transforms into apatite [68]. During setting, CPCs can be moulded in order to perfectly fit to a bone cavity, where they promote bone repair through their cell-mediated resorption and/or chemical dissolution/hydrolysis in the body fluids [69]. Further advantages of CPCs include good injectability, low cost, easy preparation and handling, and ability to be replaced by new formed bone. On the other hand, their main drawbacks are lack of macroporosity, which is needed for impregnation of biological fluids and fast bone ingrowth, and poor mechanical properties, which limit their use in load-bearing applications [70,71]. A vast number of additives, including biological and synthetic polymers, inorganic compounds, accelerators and retardants of the setting reaction, have been proposed to improve cement mechanical properties [72,73,74,75,76,77]. The non-exothermic reaction of CPCs allows loading with biological molecules and drugs, which can be added to the solid, as well as to the liquid phase [78]. In particular, CPCs have been proposed as delivery systems of growth factors, proteins, antibiotics, anti-inflammatory, anti-osteoporotic and anti-cancer agents [78,79].

The use of CPCs for the local administration of BPs represents a real challenge due to the great affinity of BPs for calcium. BPs strongly interact with the cement powder with consequent lengthening/inhibition of setting times and worsening of the mechanical properties of the cement [80,81,82,83,84].

Strategies based on the addition of BPs to the liquid phase allowed the loading of just very small amounts of drugs to α-TCP-based cements. However, these quantities were found sufficient to promote in vitro osteoblast differentiation and to inhibit osteoclastogenesis and osteoclast activity both in the case of alendronate and pamidronate [80]. Addition of gelatin to the cement composition was utilized to increase the amount of alendronate loaded into the cement, while maintaining suitable setting times, workability and proper mechanical properties for low load bearing sites [85].

Other studies utilized one of the components of the cement powder, namely calcium deficient hydroxyapatite (CDA), to load BPs through chemisorption. The inclusion of zoledronate loaded CDA in an injectable CaP matrix was demonstrated to provide a material able to improve bone micro-architecture in a rat model, as well as in the proximal femurs of osteoporotic ewes [86]. On the basis of these encouraging results, CDA was utilized to incorporate alendronate into an α-TCP-based cement formulation [81]. The resulting cement was tested in vivo in a preclinical large animal model. Implantation in osteopenic bone defects in vertebrae of adult female sheeps showed a beneficial effect on bone content and on micro-architecture of the adjacent trabecular bone. The dependence of the effect on the distance from the implant was suggested to be related to the local distribution of alendronate released from the cement [21].

**Figure 4:**
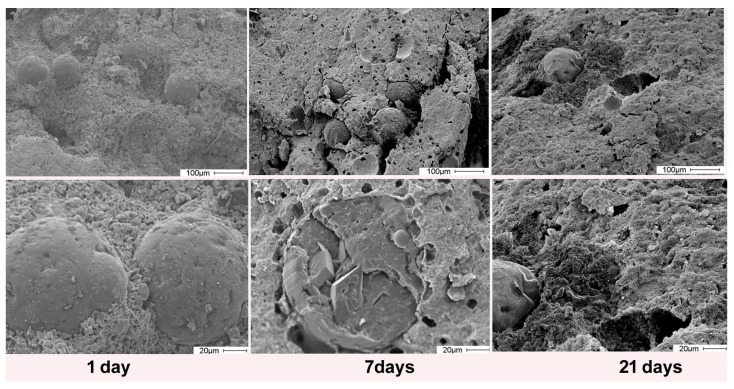
Scanning electron microscopy images of fractured surfaces of CPC samples containing lipid microspheres loaded with alendronate after different times of soaking. The microspheres embedded in the cement maintain their rounded shape for up to seven days of soaking, and then gradually lose their morphology. Modified from reference [87] Copyright (2018), with permission from Elsevier.

A recent study showed that the use of solid lipid microparticles (MPs) as carriers to enrich bone cement formulation with alendronate allowed others to achieve significantly greater alendronate content than that previously reported for a cement of similar composition [87]. Inclusion into MPs limits alendronate interaction with the cement. As a consequence, the presence of the BP has just a modest influence on setting times and mechanical properties. Moreover, this strategy ensures a sustained release of the drug for at least 21 days (Figure 4) [87].

### 4.2. Coatings

Replacing bone in high load-bearing sites requires the use of materials with suitable mechanical properties. CaPs display very good characteristics of biocompatibility and bioactivity, but poor mechanical parameters, which makes the coupling with resistant and flexible materials, such as metals, mandatory. However, metallic implants do not form mechanically stable bonds to bone tissue and coating with CaPs is considered one of the best ways to enhance the biological properties of metallic prostheses [4]. CaPs coatings can be produced through a variety of methods, some of which have been also utilized for obtaining BP functionalized CaP coatings.

One of the proposed strategies involves the deposition of a CaP layer on the metallic substrate, followed by the adsorption of the drug. A biomimetic apatitic coating can be easily produced after soaking in a Simulated Body Fluid solution or in a Dulbecco’s Phosphate Buffered Saline and calcium chloride. It was shown that pamidronate [88], as well as zoledronate [89], could be successfully adsorbed on the pre-formed coatings. XPS analysis, which was used as an effective tool for BP detection, confirmed the presence of nitrogen. Shen et al. [90] deposited HA layers on the surfaces of titanium oxide nanotubes via an alternating immersion method by soaking in a supersaturated Ca(OH)_2_ solution in order to calcify their inner surfaces, followed by alternately immersion into solutions of calcium nitrate and ammonium phosphate for 10 times. 12 h incubation in alendronate solution produced a coating that displayed a sustained release of alendronate in PBS solution even after 21 days. In vivo tests on osteoporotic rabbits demonstrated that these materials have great potential for enhancing local osseointegration compared with similar implants not containing alendronate. The good osseointegration and anti-osteoporosis properties were suggested to be due to the synergic contribution of released calcium and alendronate [90]. Among biomimetic methods of deposition, three possible methods of loading alendronate into CDHA coatings were systematically studied and compared: (I) BP aqueous solution contact with pre-formed CDHA coatings; (II) co-precipitation of BP–CDHA coatings; (III) alternative layer by layer coatings of CDHA and BP by several cycles [91]. The results indicated that the co-precipitation method yields incorporation of alendronate in the inner layers of the coatings modifying its release.

Plasma spray techniques have been greatly utilized in these kind of studies for the deposition of CaP coatings to be loaded with BP through adsorption from solution. XPS depth profiling of pamidronate-exposed HA coatings showed penetration of the BP into the ceramic coating to depths of at least 260 nm. In vitro pamidronate loaded HA exhibited a 25-fold decrease in primary osteoclast cells in comparison to unloaded HA [92]. Furthermore, bone histomorphometry indicated that locally released alendronate from a plasma-sprayed HA composite coating can significantly inhibit the osteolytic effect of particles on peri-implant bone. Alendronate released during the first week, which accounted for about a half of the absorbed BP, could inhibit osteoclast activity and induce early bone implant integration. An additional 10% of the alendronate was released gradually in the following 11 weeks, which could ensure a continuous, low dose drug concentration around the implant and benefit peri-implant bone formation [93].

It may be also interesting to compare the in vivo behaviour of different BPs adsorbed on HA coatings under the same experimental conditions. For this purpose hydroxyapatite-coated titanium implants were functionalized by immersing their plasma sprayed HA pre-coated surface in a solution of pamidronate, ibandronate, or zoledronic acid at the same concentration (1 mg/mL), and implanted in ovariectomized rat tibiae. All the three BPs showed positive effects on implant fixation in osteoporotic bone, with a rank order of zoledronic acid > ibandronate > pamidronate [94].

**Figure 5:**
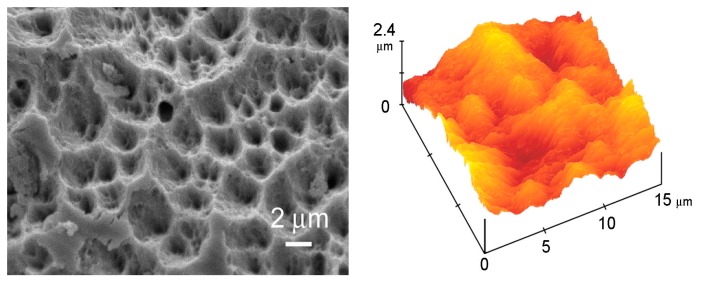
SEM micrograph of a thin film deposited from: HA containing 7 wt % of alendronate, and AFM image of the surface of a thin film deposited from HA. Reprinted from reference [96] Copyright (2009), with permission from Elsevier.

A different approach for the preparation of functionalized coatings is the deposition on a substrate of pre-synthesized functionalized CaP powders. In this case, the physical technique employed for the deposition is required to be mild enough not to damage the material. Bone-like HA nanocrystals functionalized with alendronate through physisorption have been deposited on titanium disks using the electrospray deposition method, which allowed others to obtain uniform coatings about 700 nm thick, with a maximum amount of drug of 29.5 wt %, as determined by thermogravimetric analysis. In vitro tests showed the efficacy of functionalized alendronate-HA coatings in reducing osteoclast number [95]. Matrix Assisted Pulsed Laser Evaporation technique (MAPLE) is useful for the preparation of ceramic coatings containing drugs, since it provides a gentle mechanism for transferring different compounds and allows to preserve organic material properties after transfer. The deposition of HA powders with alendronate content up to 7 wt % produced crystalline coatings with a porous-like structure (Figure 5) that showed in vitro a beneficial influence on osteoblast growth, viability and earlier differentiation whereas they inhibited osteoclast proliferation and differentiation, eventually promoting their apoptosis [96].

Deposition of thin films with variable compositions can be obtained using Combinatorial MAPLE (C-MAPLE), which yields compositional gradients by simultaneous laser vaporization of two distinct material targets. In particular, strontium and zoledronate HA could be co-deposited obtaining crystalline coatings with a tunable content of strontium up to about 8 atom % and zoledronate up to about 7 wt %. The coatings showed a granular morphology, with grain dimensions of the order of tens of nanometers, without significant variation as a function of composition [97]. Osteoblast and osteoclast behavior when co-cultured on the coatings depends on the relative content of strontium and zoledronate, so that it is possible to tailor-made suitable coatings modulating their effects in promotion of bone growth and prevention of bone resorption.

### 4.3. Scaffolds

Regenerative medicine aimed to solve the numerous problems affecting the musculoskeletal system is based on the development of artificial bone graft materials able to promote bone regeneration. In particular, ideal three-dimensional scaffolds should be osteoconductive, osteoinductive and biocompatible. In other words, they should not stimulate any toxic or adverse reaction, whereas they should promote cell attachment, proliferation and differentiation, as well as normal bone growth [98]. Porosity, both on the micro and the nano scale, is a further mandatory requirement of scaffolds for bone tissue engineering [2]. CaPs, both alone and in combination with biological and synthetic polymers, are widely employed for the preparation of 3D-porous scaffolds [99,100]. Porosity can be obtained using a variety of methods, including gas foaming, freeze drying, electrospinning, and 3D printing [101,102]. Porous scaffolds have been proposed as delivery systems for BPs [103,104,105,106,107,108,109]. Loading of the drugs can be achieved through adsorption on the pre-formed scaffold, through addition to the bulk structure as scaffold components, as well as via incorporation onto the scaffold of BP loaded liposomes [107,109,110,111]. Tarafder and Bose [107] loaded alendronate on 3D printed interconnected porous tricalcium phosphate (TCP) scaffolds and implanted as prepared scaffolds and polycaprolactone coated scaffolds in the distal femoral defects of Sprague Dawley rats. The results of the tests at 6 and 10 weeks indicated that the presence of alendronate promotes greater bone formation and reduces tartrate resistant acid phosphatase (TRAP) positive cell activity, whereas polycaprolactone allows controlled drug release.

**Figure 6:**
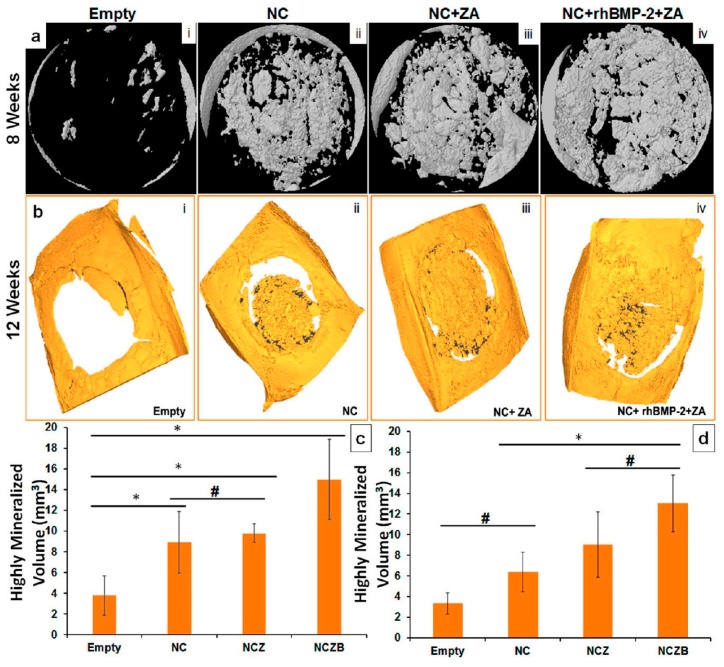
Histomorphometry analysis using micro-CT at 8 and 12 weeks. (**a**) Micro-CT images showing surface rendered 3D model images of defect site at 8 week and (**b**) Micro-CT rendered image of defect site at 12 week time point, showing the amount of highly mineralized volume present at the defect and bone material integration; (**c**,**d**) Micro-CT-based histomorphometry analysis of the defect site showing amount of highly mineralized volume at the defect site at 8 and 12 weeks time point (* *p* < 0.05; # no statistical differences between the groups). NC: Nano-HA and calcium sulfate; ZA: zoledronic acid; rhBMP-2: Bone morphogenetic protein-2. Reprinted with permission from reference [112] Copyright {2017} American Chemical Society.

Multifunctionalization of a biomimetic collagen hydroxyapatite scaffold was performed in order to provide a system for local delivery of rhBMP-2 and zoledronate. Results of implantation into the rat hind limb demonstrated a significantly higher increase in bone volume in rhBMP-2/zoledronate group compared to scaffold loaded with rhBMP-2 alone, confirming the ability of the bisphosphonate to enhance rhBMP-2 promotion of bone formation [105]. The beneficial effect of the combined presence of rhBMP-2 and zoledronate on bone regeneration was confirmed by the results of a study on a functionalized nano-HA and calcium sulfate bone substitute for cranioplasty. Implantation in a critical size defect in the calvarium of Wistar rats for 12 weeks allowed to verify that the simultaneous presence of the anabolic and anti-catabolic agents provided the highest degree of mineralization in comparison to the other groups (Figure 6) [112].

Calcium phosphate microspheres have also been proposed as sustained delivery systems of BPs. Kim et al. [113] used a water-in-oil microemulsion to load up to about 22 wt % of alendronate in situ on HA microspheres. After an initial burst release, HA dissolution was accompanied by a sustained alendronate release over 40 days. Similar significant amounts of alendronate were loaded by Lee et al. [114] on HA microspheres prepared by enzymatic decomposition followed by water-in-oil emulsification and sintering.

The presence of HA was shown to play a key role on the enhancement of alendronate encapsulation efficiency in poly(lactic-co-glycolic acid) (PLGA)-based microspheres, as well as on chitosan-HA microspheres [115,116]. PLGA/HA-alendronate microspheres displayed a sustained drug release over 30 days and an inhibitory effect on the growth of macrophages [115]. The amount of alendronate loaded on chitosan-nanoHA microspheres could reach ten times greater values (about 22 wt %) than on chitosan microspheres (about 2 wt %), and exhibited an improved drug release profile [116].

Gelatin/octacalcium phosphate core/shell microspheres were realized thanks to the great affinity of BPs for calcium. Functionalization of gelatin with alendronate improved attraction towards calcium phosphate deposition and was an essential step to promote deposition of the calcium phosphate shell. In particular, an alendronate content of 4.8 wt % provided the deposition a complete OCP porous shell, which modulated gelatin and alendronate release suggesting potential application of the microspheres as drug delivery systems [117].

## 5. Concluding Remarks

The use of calcium phosphates as delivery systems of therapeutic agents is a rapidly growing field. The subject is particularly relevant when the systemic administration of the same drug is accompanied by adverse side effects, as in the case of BPs. The studies on functionalization of calcium phosphates with BPs provide important information on the chemical and structural interactions between these drugs and calcium phosphates, which can be usefully exploited also to gain a deeper knowledge of the interaction of BPs with the inorganic phase of bone. Further improvement of the in vitro and in vivo performance of these materials can be achieved through enrichment with other bioactive agents, such as growth factors, able to enhance new bone formation. BP functionalized calcium phosphates are already proposed, alone or in combination with synthetic and biological polymers, as raw materials for the preparation of porous supports for regenerative medicine, coatings for metallic substrates and bone cements. Additional efforts aimed to increase the amount of loaded drug and to control its release could further improve the potentiality of these materials for applications in bone tissue regeneration.
